# DISIS: Prediction of Drug Response through an Iterative Sure Independence Screening

**DOI:** 10.1371/journal.pone.0120408

**Published:** 2015-03-20

**Authors:** Yun Fang, Yufang Qin, Naiqian Zhang, Jun Wang, Haiyun Wang, Xiaoqi Zheng

**Affiliations:** 1 Department of Mathematics, Shanghai Normal University, Shanghai, China; 2 Department of Mathematics, Shanghai Ocean University, Shanghai, China; 3 Department of Bioinformatics, School of Life Science and Technology, Tongji University, Shanghai, China; Cedars-Sinai Medical Center, UNITED STATES

## Abstract

Prediction of drug response based on genomic alterations is an important task in the research of personalized medicine. Current elastic net model utilized a sure independence screening to select relevant genomic features with drug response, but it may neglect the combination effect of some marginally weak features. In this work, we applied an iterative sure independence screening scheme to select drug response relevant features from the Cancer Cell Line Encyclopedia (CCLE) dataset. For each drug in CCLE, we selected up to 40 features including gene expressions, mutation and copy number alterations of cancer-related genes, and some of them are significantly strong features but showing weak marginal correlation with drug response vector. Lasso regression based on the selected features showed that our prediction accuracies are higher than those by elastic net regression for most drugs.

## Introduction

Elucidating the relationships between genetic alterations and cancer vulnerabilities is a major task for current cancer genome projects. As is known, cancers are induced by the accumulation of genetic alterations within a cell, including inherited genetic mutations chromosome translocations, and copy number alterations [1,2]. Association analysis between genetic alterations and anticancer drug sensitivity could provide new insights for biomarker discovery and drug sensitivity predictions. However, the huge diversity of different cancer types, even tumors from the same tissue, makes the above aim very challenging.

In recent years, many efforts on elucidating biomarkers for some kinds of anticancer drugs have been seen in literatures ever since the outcome of high-throughput genomic technique, and most of them are based on expression profile data. For example, Staunton et al. proposed a weighted voting classification strategy to predict a binary response (sensitive or resistant) based on the NCI-60 gene expression data [3]. Based on the same data, Riddick et al. built an ensemble regression model using Random Forest [4], and Lee et al. developed a co-expression extrapolation algorithm to infer drug signature by comparing differential gene expression between sensitive and resistant cell lines [5]. However, due to the diversity of different cancers, biomarker of a certain drug for different cancer types may be different, so other researches focused on some specific type of cancer. For example, Holleman et al. investigated gene-expression patterns in drug-resistant acute lymphoblastic leukemia cells, and found combined drug-resistance gene-expression score is significantly associated with the risk of relapse [6]. Besides gene expression, some researchers focused on the possible relationships between chemical therapy sensitivity and some epigenetic modifications such as phosphorylation and methylation. For example, Shen et al. used CpG island methylation profile to predict drug sensitivities in NCI-60 cancer cell line panel [7]. They got a list of methylation markers that predicted sensitivity to chemotherapeutic drugs, e.g., hyper-methylation of the p53 homologue p73 and associated gene silencing was strongly correlated with sensitivity to alkylating agents. Menden et al. [8] utilized cell line features including microsatellite instability status and copy number variances of 77 oncogenes as well as physicochemical properties of drugs to train a neural network model for drug sensitivity prediction. However, despite the success in finding some drug biomarkers, these kinds of methods still suffer from the limited number of samples (cell lines), compared with the large number of expression genes and chemical compounds (>100,000). So it is possible to over-estimate the gene signature for some compounds by chance.

Recently, researchers from the Broad Institute of Harvard and MIT and Sanger Institute generated a large scale genomic data set for more than 1000 human tumor cell lines, including mutation status, copy number variance, expression profile and translocation of a selected set of cancer driver genes, as well as the pharmacological profiles for a large number of anticancer drugs [9,10]. To elucidate the interaction between genomic instabilities and drug sensitivity, they first screened all genomic features and discarded all irrelevant features whose Pearson correlation coefficients (*r*) with drug response are weak (*|r|< 0*.*1*), and then applied a so-called elastic net regression to estimate sensitivity from the selected genomic instability data. Although achieving good performance for some certain drugs and samples, their model only screens features by their marginal information (referred to as SIS by [11]), and thus suffers from the following two inherent disadvantages. Firstly, genomic features, especially expression profiles, are not independent with each other but form a very complicated association structure, which could not be grasped by the SIS method. Secondly, many marginally strong features could have very high pairwise correlations, but they are jointly uncorrelated with the response and thus not helpful in the subsequent model prediction. Moreover, these ‘redundant’ features may dominant the final feature list for having higher priority to be selected by SIS than other important features.

In this work, we apply an iterative sure independence screening (ISIS) [11,12] to overcome the inherent disadvantages of SIS and further improve the prediction accuracy. Through executing SIS and lasso regression interactively, the ISIS scheme could detect combination effects of some marginally weak features with the response variable. In each iteration step, SIS is carried out first to reduce the dimension of predictors from “*p*” which is much larger than the sample size “*n*” to “*d*”, a determined positive integer smaller than “*n*”. Then “lasso” [13], a moderate dimensional method, is applied to the “*d*” predictors for further variable selection and regression fitting. ISIS computes residuals based on the model fitted using the recruited features, and then uses the residuals as the response variable to continue recruit new features. It works by iteratively performing feature selection and can overcome the aforementioned drawbacks of SIS. ISIS scheme has been widely used in many applications such as gene selection and disease classification to deal with the so-called ultrahigh dimensional feature selection [14,15]. For each drug in the CCLE dataset, we selected up to 40 features by ISIS from more than 30,000-D feature space including gene expression, copy number variance and mutation of cancer related genes. Regression results showed that our approach achieved improved accuracy for nearly all drugs compared to the elastic net model and some important marginally weak features for drug sensitivity discovery were detected.

## Material and Method

### Data sources

The cancer genomic and drug response data used in this work are available from the Cancer Cell Line Encyclopedia (CCLE). This dataset consists of a large scale of genomic data, i.e., gene expression, mutation status and copy number alteration for 947 human cancer cell lines, as well as 8-point dose-response curves for 24 chemical compounds across 479 cell lines. We used the area under dose-response curves (termed as Activity area in [9] to evaluate the sensitivity of drug to a given cell line. Compared to the IC50 and EC50, activity areas could capture the efficacy and potency of a drug simultaneously. These data allow systematical discovery of biomarkers or signatures able to characterize, classify, and prognosticate clinical behavior of human tumors.

### Feature selection through iterative sure independence screening

All genomic features including expression profile of 20069 genes, mutation status of 1667 genes and copy number status of 21217 genes are integrated as the feature vector to represent one cell line. Similar to the elastic net model, we also assume a linear model between drug response and the genomic features of a cell line. Since the number of features is much larger than the sample size, the classical methods such as ordinary least squares fail to fit the linear regression model. Also, it is assumed that only a few of the features are really associated with the drug response. So, we should first identify the features that are responsible for the response of a given drug to reduce the number of effective features. In this paper, an iterative sure independence screening (ISIS) scheme is conducted for this purpose. To avoid confusion, we hereby clarify that the “DISIS” in the paper title emphasizes the iterative sure independence screening for the prediction of drug response, while in the following content we use “ISIS” to highlight the method itself.

**Fig 1:**
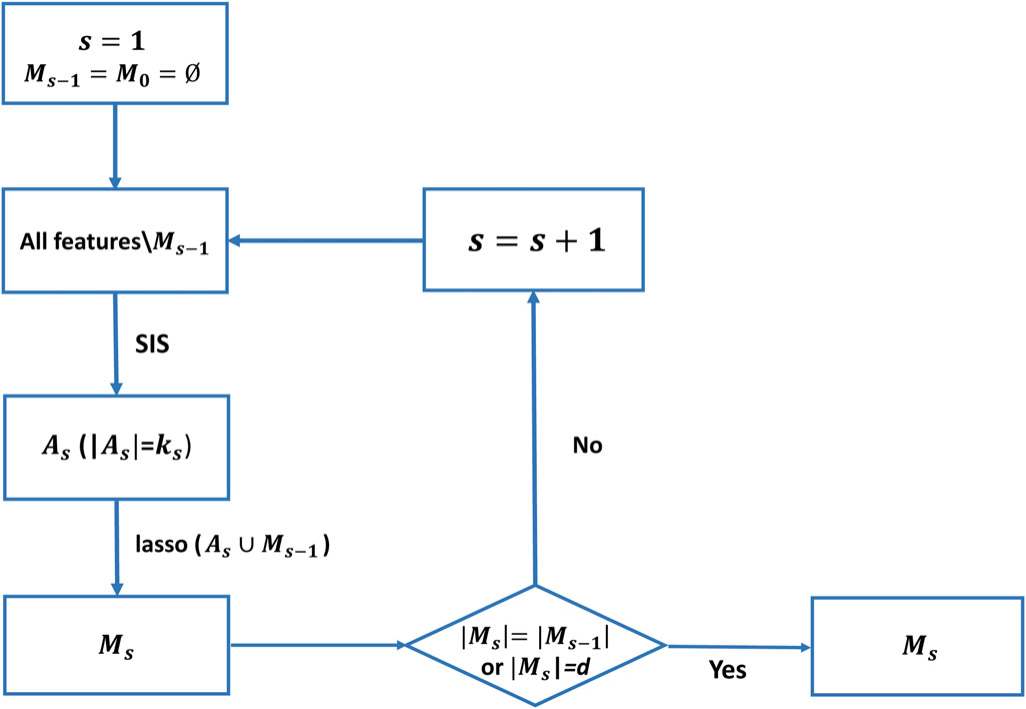
Workflow of the iterative sure independence feature screening. The sure independence feature screening (SIS) is iteratively operated on the entire feature space or residual space to get a set of candidate features, and the selected features are then input to a lasso regression to get the final effective features.

The whole procedure of ISIS is explained in Fig. 1. Assume that we aim to select *d* features from the entire feature vector of *p*-dimension for a drug D in the CCLE dataset. In the first step, we obtain *k*_1_ features by screening all features via SIS, giving the set of the indices of the recruited features *A*_1_. In more details, we use the pairwise Pearson correlation coefficients (PCCs) of features and the response of drug D as the criterion to rank all features, and the top *k*_1_ features with the highest absolute correlations are retained. Then we do the variable selection with the lasso penalty based on a linear regression model to obtain a subset *M*_1_ of *A*_1_, by minimizing the objective function

$$L(\beta_{0},\;\beta_{j,\;j\in A_{1}})\;=\;{\sum_{i\;=\;1}^{n}\left(y_{i}\;-\;\beta_{0}\;-\;\sum_{j\in A_{1}}\beta_{j}x_{ij}\right)}^{2}\;+\;\lambda \;\sum_{j\in A_{1}}\left|\beta_{j}\right|,$$

where *x*_ij_ is the *j*-th component of the feature vector *x*_i_, *y*_i_ is the response of sample *i* to drug D. *β*_0_ and *β*_j_ are lasso estimators, *n* is the number of samples and *λ* is the penalty factor for reducing the number of effective features associated with drug response. Here *M*_1_ refers to the indices set of features with the nonzero coefficients. The numbers of features in *M*_1_is denoted by |*M*_1_|. We then build the linear regression model of the response over the predictors of *M*_1_ and get the residuals. After this step, we treat the residuals as a new response and applied SIS to get the indices set *A*_2_ of *d-*|*M*_1_| features from the features of the indices {1,2,…p}\ *M*_1_. Then we use the lasso method again to minimize

$$L(\beta_{0},\beta_{j,j\in M_{1}UA_{2}})\;=\;\sum_{i=1}^{n}{\left(y_{i\;}-\;\beta_{0}\;-\;\sum_{j\in M_{1}UA_{2}}\beta_{j}x_{ij}\right)}^{2}\;+\;\lambda \sum_{j\in M_{1}UA_{2}}\left|\beta_{j}\right|\;.$$

The non-zero coefficients gives the new indices set *M*_2_ of features. The above process of feature filtering and selection is repeated until |*M*_s_| = *d* or |*M*_s_| = |*M*_s-1_|. To make sure the ISIS can be taken iteratively and not stop at the first iteration, we use the suggestion of $k_{1}=\left[\frac{2d}{3}\right]$ in [11]. We refine the estimates of coefficients at the final step by fitting the linear model of response over the features in *M*_*s*_.

According to the construction of ISIS, the residuals from the (*q*-1)-th iteration are uncorrelated with the variables selected in the (*q*-1)-th iteration. This fact could, on one hand, significantly drop the priority of those unimportant variables that are highly correlated with the response by their associations with *x_j_,j ϵ* *M*_*q*-1_, and on the other hand, could give those important predictors that are missed in the previous steps possible another chance to survive. In conclusion, two problems of simple SIS specified in the Introduction part could be overcome using ISIS. Also, we want to point out that at the last iteration of ISIS, although the lasso regression gives the coefficients estimates, due to the well accepted fact that lasso method cannot simultaneously achieve the selection consistency and optimal estimation [16,17], in this paper the ordinary least squares (OLS) is then used to get the final regression coefficient estimates based on the selected features.

### Cross-validation and evaluation criteria

The 10-fold cross validation was used to determine the parameter in our model, i.e., the number of features *d*. In order to reduce the generalization error of a machine-learning problem, the number of features should be much less than the number of samples. To this end, we constrained the number of selected features *d* not more than 40. For each *d* in {2,4,6,…,40}, we performed 10 iterations of a 10-fold cross-validation based on the ISIS scheme and the OLS method to refine the final regression coefficients estimates. So, the drug response for D of each sample was predicted 10 times against the training sets. Finally, the Pearson correlation coefficient between the true response vector and the averaged response of the 10 predicted values was used to evaluate the performance of the model. For each drug, the final *d* was selected by maximizing the Pearson correlation coefficient of the predicted values and the response observations via the 10-fold cross validation.

### T-test for the significance of regression coefficients

After *d* was determined by the cross validation, the ISIS was implemented to the whole dataset. Then the OLS was used to get the final coefficients estimates based on the linear model formed by the selected features via ISIS scheme. It is well known that in the multiple regression models, all the features explain the response (drug sensitivity) jointly, and the explanatory effect is not the simple summation of the marginal explanatory effects. It is reasonable and possible that some features may have weak marginal importance or equivalently speaking their Pearson correlation coefficients with the response are low, but become important combined with other features. We find some features whose Pearson correlation coefficients with response are weak (*|r|<0*.*1*) which are deleted by the elastic net model [9] in the beginning, but are recruited by the ISIS. The selection results by ISIS have meant that they are important. While in the consideration of the OLS used to refine the coefficients estimation, we want to find more evidence of the importance of these marginally weak features under the theory frame of OLS. To check the importance of these features, the t-test method for the significance of regression coefficient was employed. If the coefficient is significantly different from zero, it means that the feature is important jointly with other features. Thus the testing problem stated as follows was treated.

$$H_{0}:\;\beta_{j}\;=\;0;\;H_{1}:\;\beta_{j}\neq 0$$ (1)

The subscript *j* was in the set *J = {i*: *|r(x*_*i*_,*y)|<0*.*1*, *i = 1*,*…d}* where *r(x*_*i*_,*y)* represented the Pearson correlation coefficient of the *i*-th feature and the response *y*. The significance level was set to be 0.01 hereby. When the p-value of the testing is not greater than 0.01, the corresponding coefficient is thought to be significantly different from zero. The testing problem (1) is also inherently equivalent to the model testing,

$$H_{0}:y\;=\;\beta_{0}+\beta_{1}x_{1}+\ldots \beta_{j-1}x_{j-1}+\beta_{j+1}x_{j+1}{+\beta }_{d}x_{d};$$

$$H_{1}:\;y\;=\;\beta_{0}+\beta_{1}x_{1}+\ldots +\beta_{d}x_{d}.$$

So when we reject *H*_0_, it means that the model in *H*_1_ with *x*_*j*_ can explain the response better than the model in *H*_0_, and the existence of the feature *x*_j_ is meaningful.

## Results

**Fig 2:**
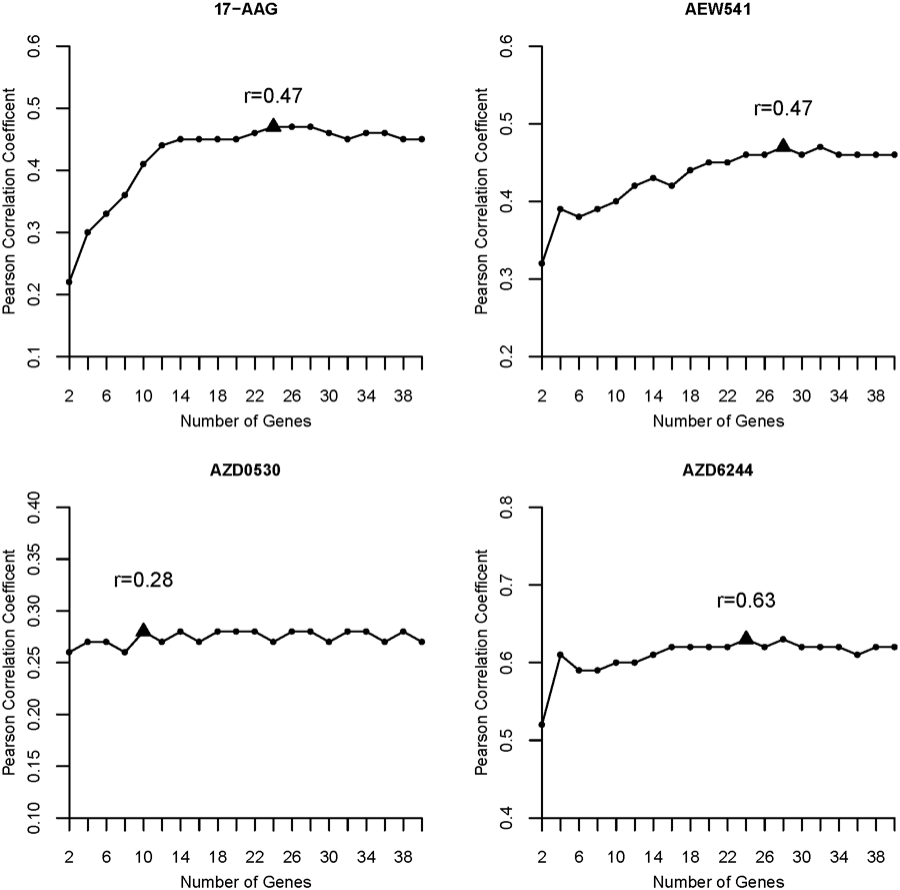
PCCs between predicted and real drug sensitivities (DS) at different numbers of recruited features.

We first explore the prediction performance for each drug of our method with respect to different top features selected by ISIS. Pearson correlation coefficients (PCCs) between real and predicted drug sensitivities (in terms of the activity area) for four example drugs are shown as Fig. 2, and results for other drugs are shown as S1–S5 Figs. We could observe that, for most drugs, the predicted PCCs do not show significant variance with the increase of selected features, that is to say, only a few number of features are enough to reflect the general pattern of the feature signature. So for each drug, we just selected less than 40 features and highlighted them by a triangle in Fig. 2.

### ISIS could remove the redundancy between identified features

The final selected features for each drug are shown in S1 Table. As aforementioned, one advantage of the ISIS method is the removal of redundancy between selected features. To demonstrate this, the mean redundancy score [18], measured by the PCC and the mutual information (MI) [19], was used to assess the redundancy between identified features. In addition, we also implemented the simple top features (STF) method by ranking the features through the marginal Pearson correlation coefficients with the drug responses, where the number of features in STF is the same as that in ISIS. The MRSs for the 24 drugs through ISIS and STF are listed in S2 Table. The mean of MRSs (PCC) by ISIS is 0.1924, whereas that by STF is 0.4963, suggesting that the feature redundancy is significantly removed by ISIS compared with STF (p-value<10^-8^ by paired t-test). Moreover, if measured by MI, the means of MRSs by ISIS and STF for the 24 drugs are 0.0753 and 0.1394 respectively, also showing significant difference by t-test (p-value<10^-3)^. All above results confirms that ISIS could remove the redundancy between selected features.

### ISIS could detect weak features that jointly correlate to drug response

It is shown that a lot of selected features (S1 Table) are consistent with the literature reports and have significant overlap with that by elastic net regression. Also similar to elastic net regression, most selected features by our algorithm are gene expression data rather than mutation and copy number alteration status, which is expected since expression profile constitutes the majority of original feature source. Among the selected features, a lot of them are well-accepted indicators for drug response. For example, the selected mutation features for AZD6244 and PD.0325901 include BRAF and NRAS, which are known to be the predictor of sensitivity to MEK inhibitors. Mutation of BRAF and EGFR are also ranked as the top feature for PLX4720 (BRAF inhibitor) and Erlotinib (EGFR inhibitor), respectively. These strong features are also successfully selected as drug response predictors by elastic net regression (ENR) [9].

Besides the above-mentioned genes, we also detected many other cancer related genes which are deleted by Barretina et al. 2012 due to their very small marginal correlations with drug response, but are significant for the regression model according to the t-test of regression coefficients (S1 Table). Interestingly, many of them are biologically relevant with the tumorigenesis. For example, the expression of GREM2 and LGI1 are predicted as strong features with weak marginal effect for an MEK inhibitor AZD6244, their correlations with drug responses are only 0.017 and 0.094, respectively. It is found that the later gene, LGI1, is predominantly expressed in neural tissues and its expression is reduced in low grade brain tumors and significantly reduced or absent in malignant gliomas [20,21]. The BCL2L13 (official name BCL1) is a selected weak-marginal gene expression feature for an Src inhibitor AZD0530. This gene has three alternative splicing results and one of them (isoform 1) could enhance cell survival by inhibiting apoptosis while the other two gene products promote apoptosis and are death-inducing [22]. RASSF2 and RASA3 are also weak-marginal features for Erlotinib and RAF265, respectively. Both genes were reported to have potential regulatory impact on RAS, where RASSF2 seems to modulate some of the growth inhibitory response mediated by RAS and may serve as tumor suppressor [23,24], and RASA3 may act as a suppressor of RAS function and thereby controls the cellular proliferation and differentiation [25].

### ISIS achieved higher Pearson correlation coefficients with real sensitivities for most drugs in CCLE dataset

Based on the selected features in the above procedure, we performed 10 iterations of 10-fold cross-validation to validate our algorithm. In detail, all cell lines treated by one drug were split into roughly 10 groups, and one of them was treated as the test dataset to measure the consistence of the predicted drug responses (ActiveArea) with their real values, with the model and parameters trained by the rest 9 groups. The above procedure was repeated for 10 times, and the average predicted drug responses were compared to the real one to get the Pearson correlation coefficients.

**Fig 3:**
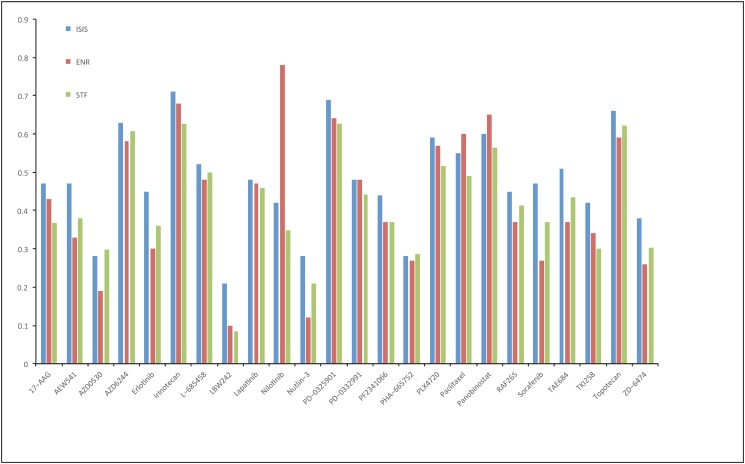
Comparison of ISIS with elastic net regression and simple top features method in drug sensitivity prediction. Pearson correlation coefficients between predicted (mean of 10 iterations of 10-fold cross-validations) and real drug sensitivities by ISIS (blue), Elastic net (red), simple top features (green).

In theory, ISIS could improve the prediction of drug responses by the combination effect of the marginally weak features and other strong features. To verify this, we compared the prediction results of ISIS with Elastic net regression (ENR) and the simple top features (STF) method, with the same numbers of selected features as ISIS. The Pearson correlation coefficients between real and predicted drug responses by ISIS, ENR and STF are reported in S3 Table and showed by a bar chart in Fig. 3. We could conclude that our prediction was slightly better than ENR and STF for most drugs. For example, 19 of 24 drugs in CCLE dataset achieve the Pearson correlations higher than 0.4 by ISIS, but the number is merely 12 for ENR. Three drugs in Fig. 3, i.e., AZD0530, LBW242 and Nutlin-3, are shown to have very weak correlations (< 0.2) of the real and predicted drug sensitivities by ENR (Supplementary Figure 10 of paper [9]), but all of them are predicted to have higher correlations than 0.2 by our algorithm. In addition, the predicted correlations by ISIS are better than those by ENR, with the paired t-test statistic 2.2658 (p-value = 0.0166). The paired t-test statistic increases to 4.9925 (p-value<10^-4^) if the exception of Nilotinib is removed. Also, ISIS gives higher predicted correlations than STF (paired t-test statistic 7.8938, p-value<10^-7^), and the performance of ENR and STF is comparable as expected, with p-value = 0.6227 by a two-sided t-test. Similar t-test results are obtained when Nilotinib is removed. Combining all of these results, we summarized that the ISIS method could identify some marginally weak features, and beat the other two methods through the combination effects of all detected features including the weak ones.

**Fig 4:**
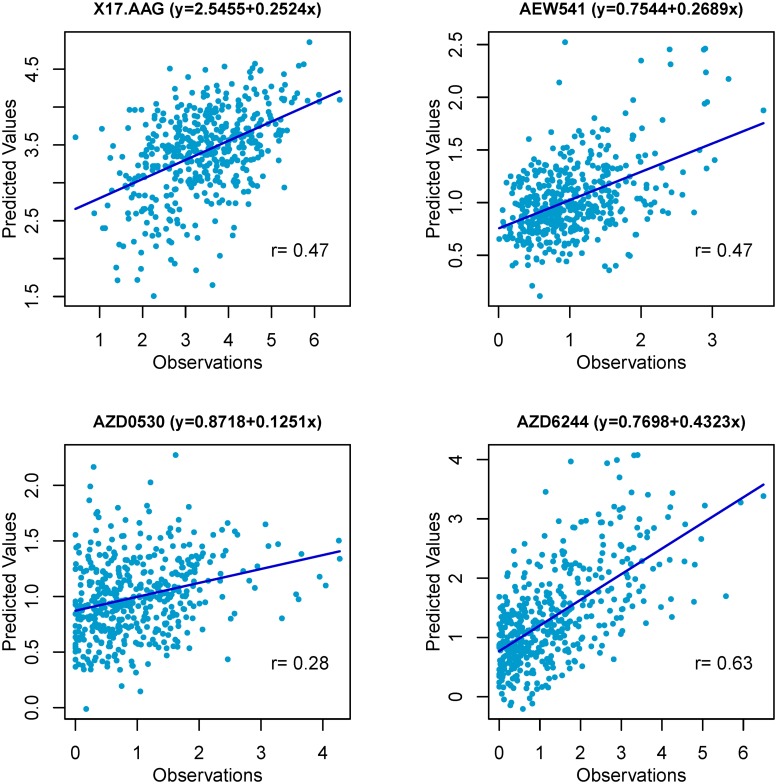
Scatter plots of the true and predicted sensitivities for some drugs.

**Fig 5:**
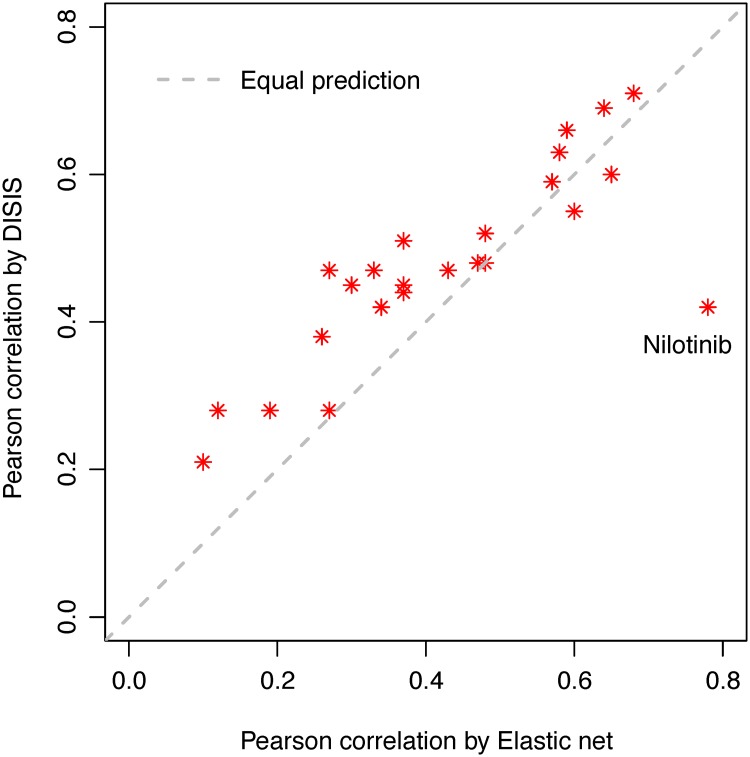
Consistence between correlations of true and predicted drug responses by elastic net and ISIS. Dotted line indicates equal predictions.

Scatter plots of the observed and predicted responses for some typical drugs are shown as Fig. 4. We could conclude from these examples that the resulting correlation was fairly reasonable and not overestimated by a few outliers. Moreover, our predictions were in great consistence with those by ENR model, given the overall correlation of 0.81 (Fig. 5). In particular, if we discarded the only one outlier, Nilotinib, the overall Pearson correlation increased from 0.81 to 0.94. As is known, Nilotinib is a special compound for treating chronic myelogenous leukemia (CML) [26], which was successfully selected as the strongest feature for sensitivity of Nilotinib according to the CCLE paper [9]. So it is reasonable that this top feature dominated the model building and prediction, and brought a high prediction correlation by ENR. While we did not take this lineage information into consideration since we focused only on mining genomic information to explain the drug sensitivity. Except this only outlier, ISIS brought much higher predicted correlations using fewer features than ENR for most drugs, which confirmed the efficiency of ISIS in feature selection.

## Discussion and Conclusion

Inference of drug response with respect to large scale of genomic data including gene expression, copy number alteration and mutation status of cancer-related genes is a very fundamental problem in research of individual medicine. However, the huge number of genomic features compared with the relatively limited number of samples makes it an illness problem. To make full use of the marginally weak features with the drug response vector and further improve the prediction accuracy, in this paper, we used an iterative sure independence screening feature selection scheme and lasso regression to the prediction of drug response based on CCLE data. By cross validation on CCLE dataset, we reported that our algorithm could not only find many strong biomarkers which were reported in previous literatures, but also detect many marginally weak genomic features (mostly are gene expression profile) that are shown to have strong commination effects to drug response. Based on the selected features, we conducted the linear model fitting to refine the regression coefficients estimates and get the Pearson correlation coefficients between real and predicted drug sensitivities. We found that, even using only very small number of features (less than 40), our algorithm still got much higher correlations than the elastic net regression.

Additionally, we want to point out that the results of the paper could be further improved from other directions. For example, optimal features could be selected by a so called ‘minimum redundancy and maximum relevance’ scheme [27], or a novel optimization criterion which simultaneously minimized the number of selected features and cross validation error [28]. In our paper, we used cross validation to select the tuning parameter λ in the objective function of lasso, under the assumption that λ with the smallest prediction error by cross validation is the optimal parameter. However, selecting parameter by cross validation is actually not a direct way to optimize the prediction, since the objective function of lasso is the regression fitting error with penalty function added, not the prediction error. So it is helpful to consider optimizing the cross validation error using the above two strategies.

## Supporting Information

S1 Table Selected features for 24 drugs and their marginal Pearson correlation coefficients with drug sensitivities. (XLSX)

S2 Table Mean redundancy score for 24 drugs by ISIS and STF. (XLSX)

S3 Table Pearson correlation coefficients of predicted and real drug sensitivities by ISIS, ENR and STF (10-fold cross validation). (XLSX)

S1 Fig Pearson correlation coefficients of predicted and real drug sensitivities at different numbers of recruited features for drugs Erlotinib, Irinotecan, L-685458 and LBW242. (PDF)

S2 Fig Pearson correlation coefficients of predicted and real drug sensitivities at different numbers of recruited features for drugs Lapatinib, Nilotinib, Nutlin-3 and PD-0325901. (PDF)

S3 Fig Pearson correlation coefficients of predicted and real drug sensitivities at different numbers of recruited features for drugs PD-0332991, PF2341066, PHA-665752 and PLX4720. (PDF)

S4 Fig Pearson correlation coefficients of predicted and real drug sensitivities at different numbers of recruited features for drugs Paclitaxel, Panobinostat, RAF265 and Sorafenib. (PDF)

S5 Fig Pearson correlation coefficients of predicted and real drug sensitivities at different numbers of recruited features for drugs TAE684, TKI258, Topotecan, and ZD-6474. (PDF)

S1 Dataset Drug response data. (XLSX)

S2 Dataset Mutation data. (TXT)
