# Supplementary figures and images for: DISIS: Prediction of Drug Response through an Iterative Sure Independence Screening
Source: PLoS One. 2015 Mar 20;10(3):e0120408. doi: 10.1371/journal.pone.0120408 (PMC4368776; doi:10.1371/journal.pone.0120408)

**Erlotinib**

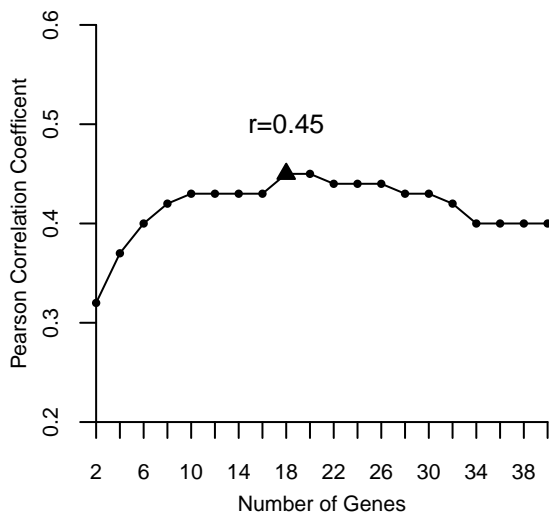

**Irinotecan**

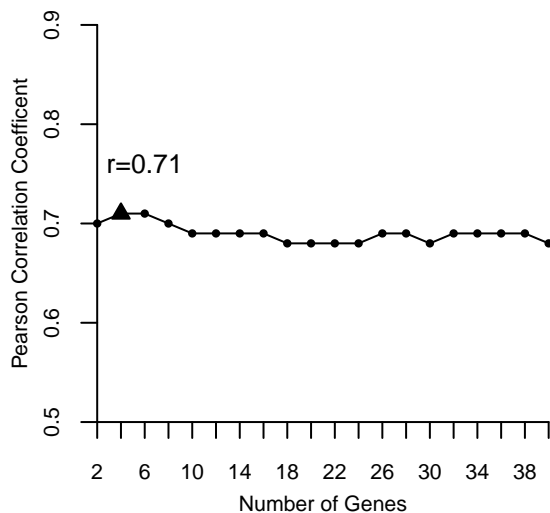

**L-685458**

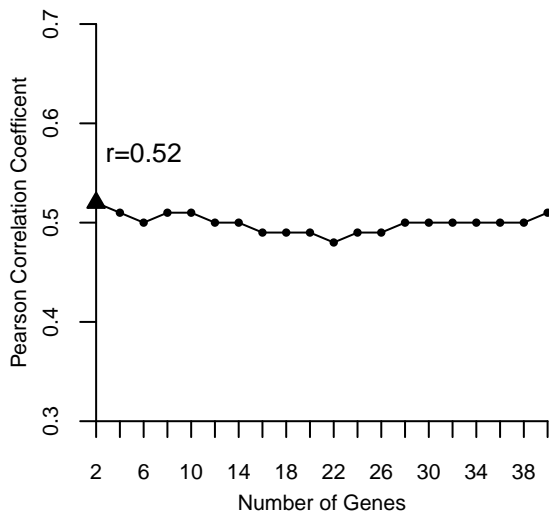

**LBW242**

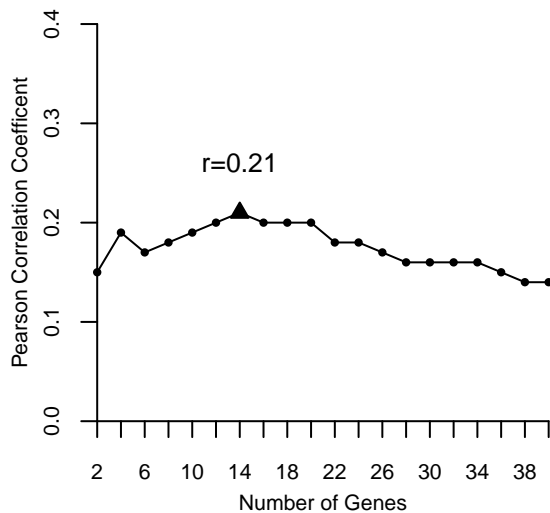

Supplement: S1 Fig — (PDF) [file pone.0120408.s004.pdf]

**Lapatinib**

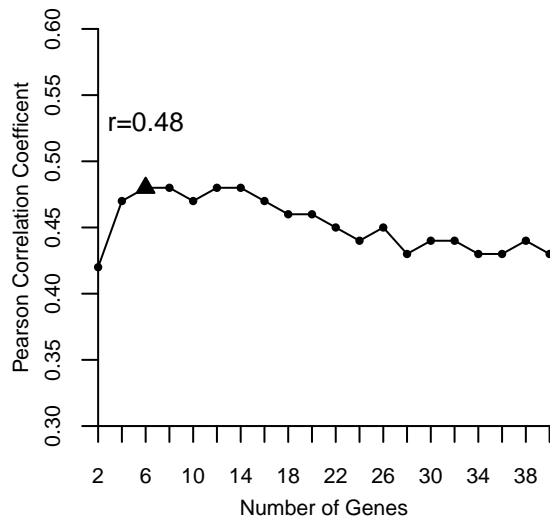

**Nilotinib**

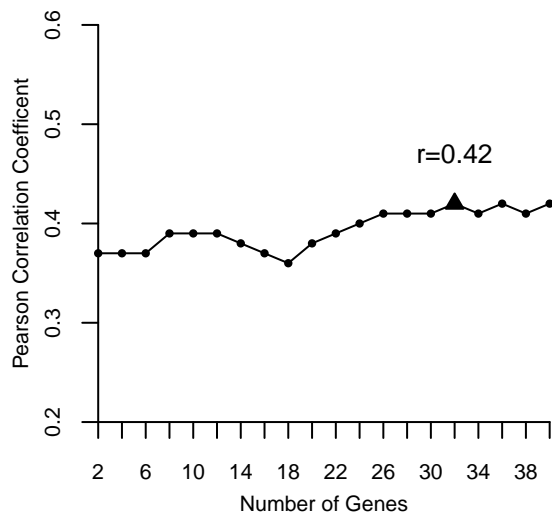

**Nutlin-3**

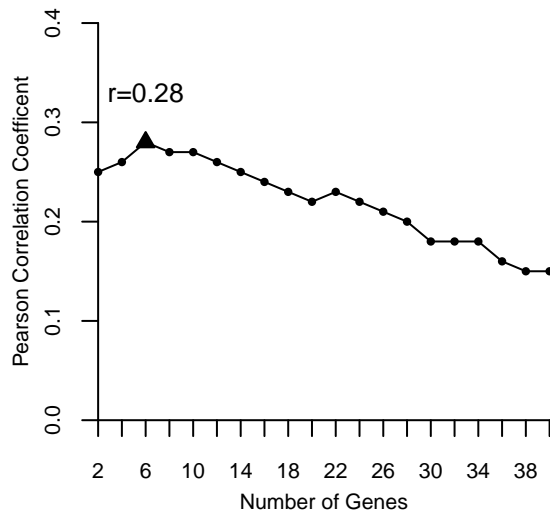

**PD-0325901**

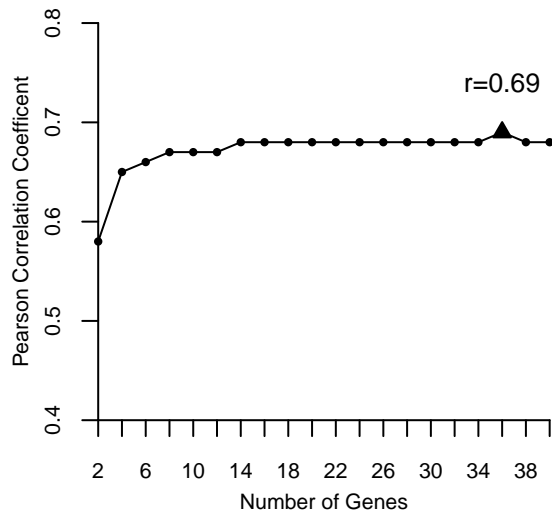

Supplement: S2 Fig — (PDF) [file pone.0120408.s005.pdf]

**PD-0332991**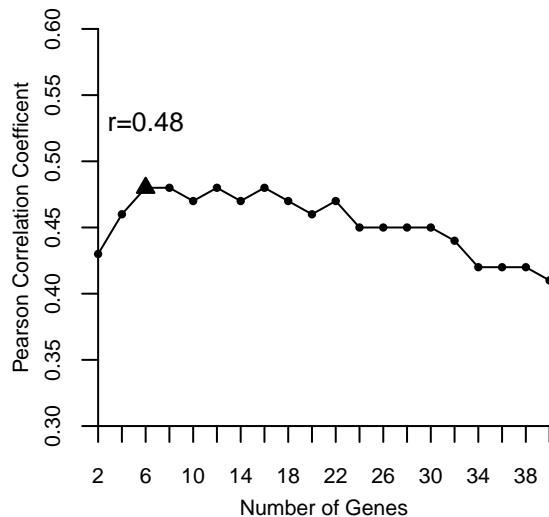**PF2341066**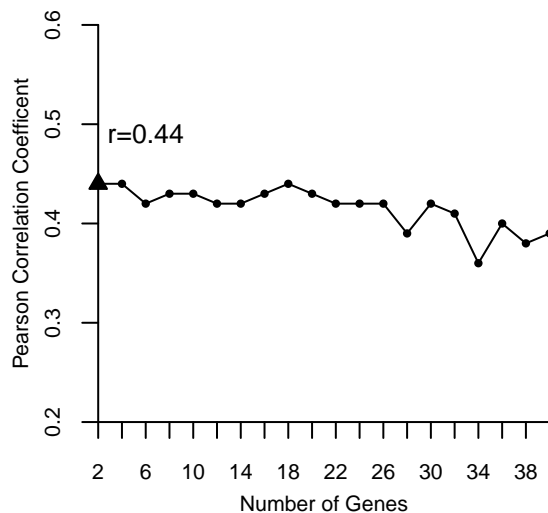**PHA-665752**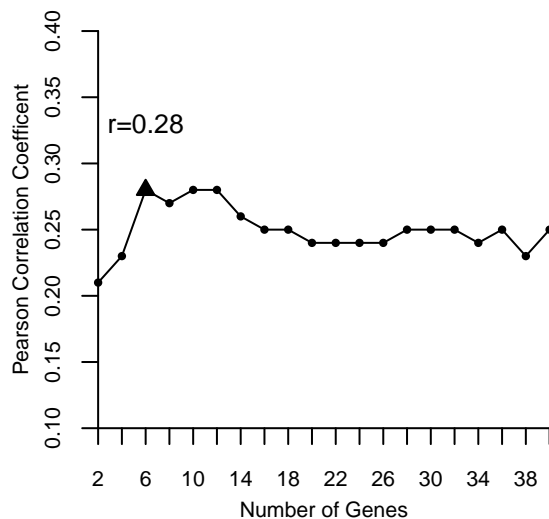**PLX4720**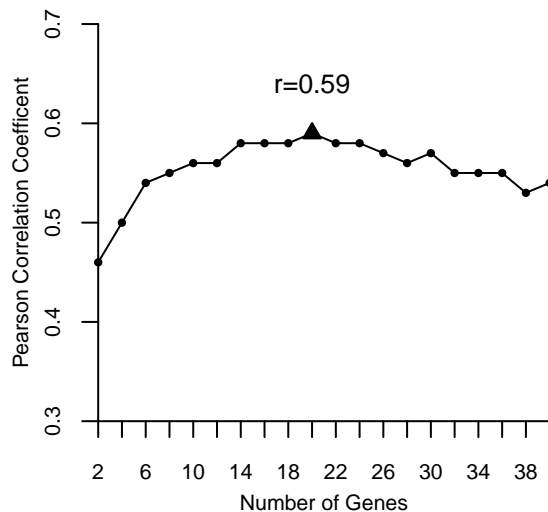

Supplement: S3 Fig — (PDF) [file pone.0120408.s006.pdf]

**Paclitaxel**

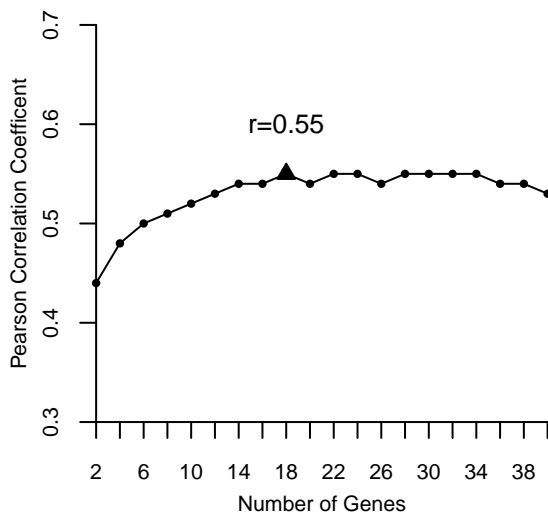

**Panobinostat**

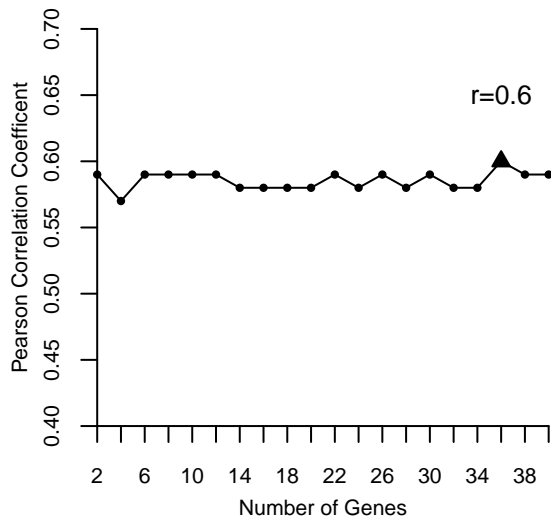

**RAF265**

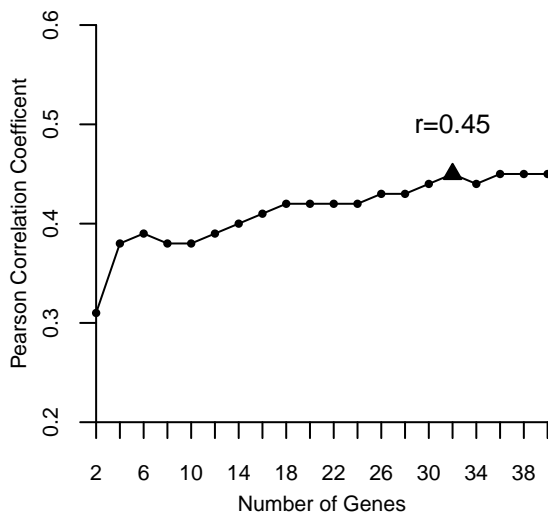

**Sorafenib**

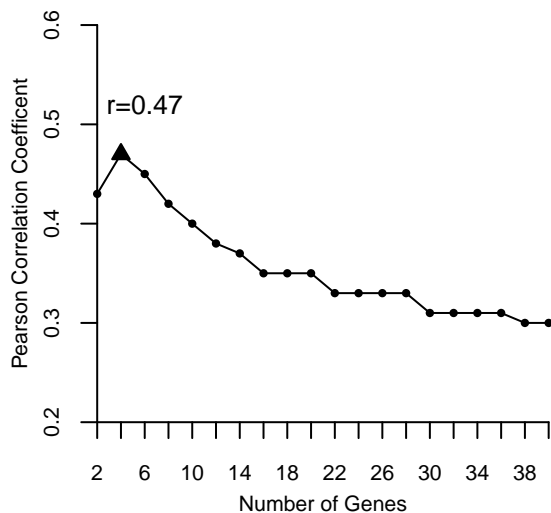

Supplement: S4 Fig — (PDF) [file pone.0120408.s007.pdf]

**TAE684**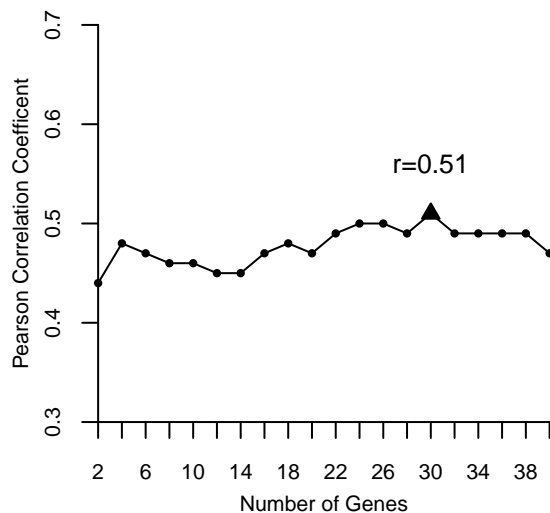**TKI258**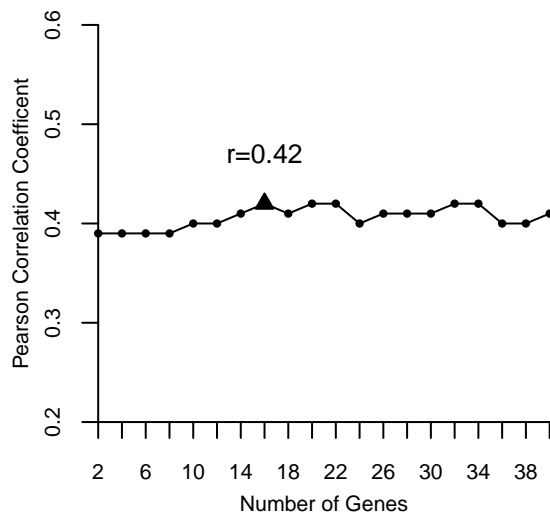**Topotecan**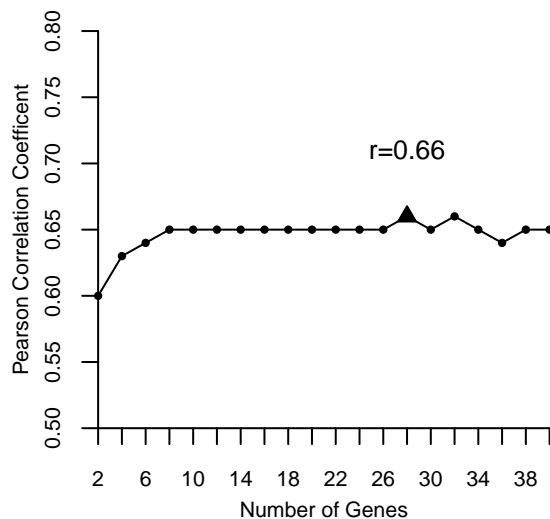**ZD-6474**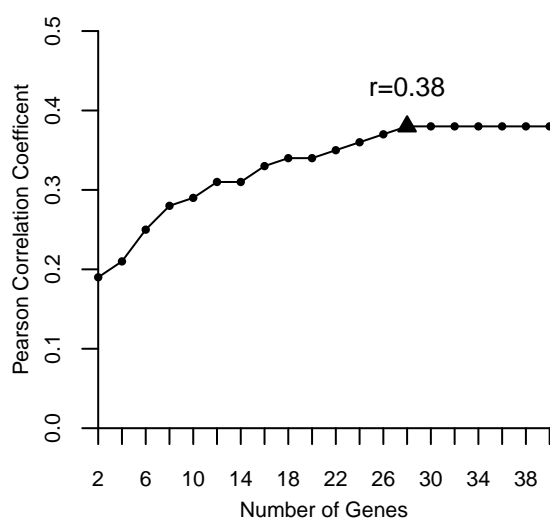

Supplement: S5 Fig — (PDF) [file pone.0120408.s008.pdf]
